# Diagnostic Utility of Platelet Indices in Differentiating Hypoproductive and Hyperdestructive Thrombocytopenia: A Cross-Sectional Study From North India

**DOI:** 10.7759/cureus.114203

**Published:** 2026-08-09

**Authors:** Reetu Choudhary, Prashant Choudhary, Shiv Prakash Sharma, Yogesh K Gupta, Hema Udawat

**Affiliations:** 1 Pathology, Rajasthan University of Health Sciences College of Medical Sciences (RUHS-CMS), Jaipur, IND; 2 Preventive Medicine, Rajasthan University of Health Sciences College of Medical Sciences (RUHS-CMS), Jaipur, IND; 3 Pathology and Laboratory Medicine, Rajasthan University of Health Sciences College of Medical Sciences (RUHS-CMS), Jaipur, IND

**Keywords:** mean platelet volume, plateletcrit, platelet distribution width, platelet large cell ratio, roc curve, thrombocytopenia

## Abstract

Background: Thrombocytopenia may result from either decreased platelet production (hypoproductive) or increased peripheral platelet destruction (hyperdestructive), and distinguishing between these mechanisms is essential for appropriate clinical management. Bone marrow examination remains the reference standard but is invasive and not always feasible. Platelet indices generated by automated hematology analyzers may provide a simple, non-invasive alternative for the initial evaluation of thrombocytopenia.

Methods: This hospital-based cross-sectional study included 150 patients with mild to moderate thrombocytopenia (platelet count 50-<150 × 10³/µL) attending a tertiary care teaching hospital in North India between 2024 and 2025. Platelet count, mean platelet volume (MPV), platelet distribution width (PDW), plateletcrit (PCT), and platelet large cell ratio (P-LCR) were measured using automated hematology analyzers. Patients were classified as having hyperdestructive (n = 114) or hypoproductive (n = 36) thrombocytopenia based on clinical evaluation and relevant laboratory investigations. Receiver operating characteristic (ROC) curve analysis was performed to evaluate the diagnostic performance of platelet indices.

Results: MPV, PDW, PCT, and P-LCR were significantly higher in patients with hyperdestructive thrombocytopenia than in those with hypoproductive thrombocytopenia (all p < 0.01). PDW (AUC = 0.747) and MPV (AUC = 0.745) demonstrated the best diagnostic performance. MPV showed the highest specificity (83.3%) at a cut-off value of 12.15 fL, whereas PCT demonstrated the highest sensitivity (78.1%) but relatively low specificity (38.9%). Plateletcrit showed a moderate positive correlation with platelet count (r = 0.464, p < 0.001).

Conclusions: MPV and PDW demonstrated good diagnostic performance in differentiating hyperdestructive from hypoproductive thrombocytopenia and may serve as useful adjunctive markers during the initial evaluation of thrombocytopenic patients. As routinely available parameters obtained from complete blood count analysis, these indices may support clinical decision-making when interpreted alongside clinical and laboratory findings. Further multicenter studies are needed to validate these findings and establish standardized diagnostic cut-off values.

## Introduction

Thrombocytopenia, defined as a platelet count below 150 × 10³/µL, is a common hematological abnormality encountered in routine clinical practice. It may result from either decreased platelet production (hypoproductive thrombocytopenia) or increased peripheral platelet destruction (hyperdestructive thrombocytopenia), two mechanisms that differ considerably in their underlying pathology, management, and prognosis [1,2].

Accurate differentiation between these mechanisms is essential because treatment strategies vary according to the underlying cause. Bone marrow examination remains the reference standard for distinguishing hypoproductive from hyperdestructive thrombocytopenia; however, it is invasive, time-consuming, costly, and may not be appropriate as an initial investigation for every patient [3]. Therefore, there is considerable interest in identifying simple, rapid, and noninvasive laboratory markers to aid in the initial evaluation of thrombocytopenia.

Automated hematology analyzers routinely provide platelet indices such as mean platelet volume (MPV), platelet distribution width (PDW), plateletcrit (PCT), and platelet large cell ratio (P-LCR) without additional cost or blood sampling [4,5]. These parameters reflect platelet size, heterogeneity, and total platelet mass and may help distinguish peripheral platelet destruction from impaired platelet production. Previous studies have reported variable diagnostic performance of these indices, and the proposed cut-off values differ across populations and disease spectra [6-9].

Evidence from North India regarding the diagnostic utility of platelet indices remains limited. Therefore, this study was undertaken to evaluate the diagnostic performance of MPV, PDW, PCT, and P-LCR in differentiating hypoproductive and hyperdestructive thrombocytopenia among patients with mild to moderate thrombocytopenia using receiver operating characteristic (ROC) curve analysis.

## Materials and methods

Study design and setting

This hospital-based cross-sectional study was conducted in the Department of Pathology, RUHS Medical College and Hospital, Jaipur, Rajasthan, India, between 2024 and 2025. The study included patients presenting with mild to moderate thrombocytopenia who underwent complete blood count evaluation during the study period. The study was designed and reported in accordance with the Strengthening the Reporting of Observational Studies in Epidemiology (STROBE) guidelines [10].

Study participants

The inclusion and exclusion criteria are summarized in Table 1. A consecutive sampling technique was adopted, and all eligible patients during the study period were enrolled after obtaining written informed consent.

**Table 1:** Inclusion and exclusion criteria.

Inclusion criteria	Exclusion criteria
Patients with platelet counts ranging from 50 × 10³/µL to <150 × 10³/µL	Platelet count <50 × 10³/µL
Patients presenting with mild to moderate thrombocytopenia	Patients receiving antiplatelet medications
Patients providing written informed consent	Clotted blood samples
Consecutively enrolled during the study period	Hemolyzed samples
	Inadequately collected samples
	Improperly stored samples
	Samples with delayed transport to the laboratory

Sample size

The sample size was estimated using the pooled standard deviation of PDW reported by Vidyadhar [7]. Assuming a 95% confidence level and an absolute precision of 0.1 fL, the minimum required sample size was calculated as 139 participants. To compensate for potential exclusions and incomplete data, 150 patients were enrolled.

Laboratory analysis

Approximately 2 mL of peripheral venous blood was collected in dipotassium ethylenediaminetetraacetic acid (K₂EDTA) vacutainers using standard aseptic precautions. Samples were analyzed within six hours of collection using Sysmex XN-1000 or Sysmex XT-4000i automated hematology analyzers (Sysmex Corporation, Kobe, Japan), which employ electrical impedance, optical light scattering, and fluorescence flow cytometry for hematological analysis.

Platelet parameters recorded included PLT, MPV, PDW, PCT, and P-LCR. Peripheral blood smear examination was performed in all cases to verify platelet morphology and exclude pseudothrombocytopenia. Additional investigations, including bone marrow examination, vitamin B12 and folate estimation, flow cytometry, serological tests, coagulation profile, and ultrasonography, were performed whenever clinically indicated to establish the underlying diagnosis.

Classification of thrombocytopenia

Patients were categorized as having either hypoproductive or hyperdestructive thrombocytopenia based on comprehensive clinical evaluation, peripheral smear findings, bone marrow examination (where indicated), and relevant laboratory and radiological investigations.

Hyperdestructive thrombocytopenia included disorders characterized by increased peripheral platelet destruction or consumption, whereas hypoproductive thrombocytopenia comprised conditions associated with impaired bone marrow platelet production.

Statistical analysis

Data were analyzed using IBM SPSS Statistics for Windows, Version 23.0 (IBM Corp., Armonk, NY, USA). Continuous variables are presented as mean ± standard deviation (SD), while categorical variables are presented as frequency and percentage.

Comparisons between the two study groups were performed using the independent-samples Student's t-test for continuous variables. Receiver operating characteristic (ROC) curve analysis was performed to evaluate the diagnostic performance of platelet indices. The optimal cut-off value for each parameter was determined using the Youden index. Sensitivity, specificity, positive predictive value (PPV), negative predictive value (NPV), and area under the ROC curve (AUC) with 95% confidence intervals were calculated. Pearson correlation analysis was performed to evaluate the relationship between platelet count and platelet indices. A two-sided p value of <0.05 was considered statistically significant.

Ethical considerations

The study protocol was approved by the Rajasthan University of Health Sciences College of Medical Sciences (RUHS-CMS), Jaipur (Approval No. RUHS-CMS/Ethics Comm./2024/416, dated May 1, 2025). The study was conducted in accordance with the ethical principles of the Declaration of Helsinki. Written informed consent was obtained from all participants or their legally authorized representatives before enrollment.

## Results

Baseline characteristics

A total of 150 patients with mild to moderate thrombocytopenia were enrolled in the study. The mean age was 33.24±18.16 years (range: 1-80 years). Of these, 92 (61.3%) were male patients, and 58 (38.7%) were female patients.

Based on comprehensive clinical and laboratory evaluation, 114 patients (76.0%) were classified as having hyperdestructive thrombocytopenia and 36 patients (24.0%) as having hypoproductive thrombocytopenia. Among the hyperdestructive group, the most common etiologies were dengue fever (79 patients, 69.3%), scrub typhus (9 patients, 7.9%), and chronic liver disease (8 patients, 7.0%). Among the hypoproductive group, the predominant causes were acute leukemia (18 patients, 50.0%) and megaloblastic anemia (17 patients, 47.2%).

Comparison of platelet indices between groups

All platelet indices showed statistically significant differences between the two groups (Table 2).

**Table 2:** Comparison of hematological parameters between hyperdestructive and hypoproductive thrombocytopenia. TLC: total leukocyte count; PCT: plateletcrit; MPV: mean platelet volume; PDW: platelet distribution width; P-LCR: platelet large cell ratio; SD: standard deviation. Values are presented as mean ± standard deviation (SD). P values were calculated using the independent-samples Student's t-test.

Parameter	Hyperdestructive (n=114)	Hypoproductive (n=36)	t-value	p-value
Hemoglobin (g/dL)	11.02 ± 2.76	6.89 ± 4.86	5.12	<0.001
TLC (×10³/µL)	7.06 ± 7.79	34.19 ± 58.89	2.89	0.001
Platelet count (×10³/µL)	73.87 ± 20.41	65.86 ± 15.48	2.17	0.032
PCT (%)	0.07 ± 0.03	0.06 ± 0.02	2.95	0.004
MPV (fL)	12.45 ± 1.12	11.39 ± 1.37	4.69	<0.001
PDW (fL)	17.80 ± 3.24	14.67 ± 3.68	4.98	<0.001
P-LCR (%)	38.63 ± 6.11	35.00 ± 8.65	2.82	0.006

MPV was significantly elevated in the hyperdestructive group (12.45±1.12 fL) compared to the hypoproductive group (11.39±1.37 fL; p<0.001). Similarly, PDW was higher in hyperdestructive thrombocytopenia (17.80±3.24 fL vs. 14.67±3.68 fL; p<0.001). P-LCR also demonstrated a significant difference (38.63±6.11% vs. 35.00±8.65%; p=0.006). PCT, while significantly different between groups (0.07±0.03% vs. 0.06±0.02%; p=0.004), showed more overlap between the two categories.

Diagnostic performance and ROC analysis

The diagnostic performance of platelet indices is summarized in Table 3.

**Table 3:** Diagnostic performance of platelet indices at optimal cut-off points. AUC: area under the receiver operating characteristic curve; PPV: positive predictive value; NPV: negative predictive value; MPV: mean platelet volume; PDW: platelet distribution width; PCT: plateletcrit; P-LCR: platelet large cell ratio. Receiver operating characteristic (ROC) curve analysis was performed to evaluate the diagnostic performance of platelet indices in differentiating hyperdestructive from hypoproductive thrombocytopenia. Optimal cut-off values were determined using the Youden index.

Parameter	Cut-off	AUC	Sensitivity (%)	Specificity (%)	PPV (%)	NPV (%)
Platelet count (×10³/µL)	63.50	0.625	58.8	63.9	62.11	60.95
MPV (fL)	12.15	0.745	63.2	83.3	78.75	69.17
PDW (fL)	16.00	0.747	68.0	75.0	71.12	70.09
PCT (%)	0.055	0.620	78.1	38.9	55.71	63.33
P-LCR (%)	35.10	0.629	77.2	47.2	62.00	67.14

MPV demonstrated the highest specificity (83.3%) and PPV (78.75%) at a cut-off of 12.15 fL, with an AUC of 0.745. PDW showed comparable diagnostic performance with an AUC of 0.747, sensitivity of 68.0%, and specificity of 75.0% at a cut-off of 16.00 fL. PCT had the highest sensitivity (78.1%) but the lowest specificity (38.9%) among all indices, with an AUC of 0.620. P-LCR showed moderate performance with 77.2% sensitivity and 47.2% specificity at a cut-off of 35.10%.

Correlation analysis

Correlation between platelet indices and platelet count is presented in Table 4.

**Table 4:** Correlation of platelet indices with platelet count. PCT: plateletcrit; MPV: mean platelet volume; PDW: platelet distribution width; P-LCR: platelet large cell ratio. Pearson correlation analysis was performed to evaluate the relationship between platelet count and platelet indices. Correlation coefficients (r) indicate the strength and direction of the linear association. A two-sided p value <0.05 was considered statistically significant.

Parameter	Pearson correlation coefficient (r)	p-value
PCT	0.464	<0.001
MPV	0.267	0.001
PDW	0.335	<0.001
P-LCR	-0.042	0.611

PCT showed a fair positive correlation with platelet count (r=0.464, p<0.001). PDW demonstrated a weak positive correlation (r=0.335, p<0.001), while MPV showed a weak positive correlation (r=0.267, p=0.001). P-LCR showed no significant correlation with platelet count (r=-0.042, p=0.611).

When analyzed separately by group, PCT maintained a fair correlation with platelet count in both hyperdestructive (r=0.425, p<0.001) and hypoproductive (r=0.554, p<0.001) subgroups, suggesting that PCT is the platelet index most consistently associated with platelet count regardless of the underlying mechanism.

## Discussion

The present study evaluated the diagnostic performance of platelet indices in differentiating hypoproductive from hyperdestructive thrombocytopenia among patients with mild to moderate thrombocytopenia. MPV and PDW demonstrated the best overall diagnostic performance, with significantly higher values in patients with hyperdestructive thrombocytopenia. Among the evaluated parameters, MPV showed the highest specificity, whereas PCT demonstrated the highest sensitivity but relatively poor specificity, indicating that MPV and PDW may be more useful as diagnostic markers in routine clinical practice.

The observed increase in MPV and PDW in hyperdestructive thrombocytopenia is biologically plausible. Increased peripheral platelet destruction stimulates compensatory thrombopoiesis, resulting in the release of larger and younger platelets into the circulation. Consequently, platelet size and size variability increase, leading to higher MPV and PDW values compared with disorders characterized by impaired platelet production [8].

Our findings are consistent with those of Negash et al., who reported that MPV and P-LCR demonstrated good diagnostic accuracy in distinguishing immune thrombocytopenia from hypoproductive thrombocytopenia [9]. Similarly, Ntaios et al. observed that increased MPV and PDW were useful indicators of peripheral platelet destruction [11]. Saran et al. also reported significantly higher platelet indices in hyperdestructive thrombocytopenia, supporting the role of these readily available hematological parameters in routine clinical evaluation [12]. Although the optimal cut-off values differed slightly across studies, these variations are likely attributable to differences in study populations, disease spectrum, analyzer platforms, and laboratory methodologies [13].

Among the evaluated platelet indices, PDW and MPV demonstrated the highest areas under the ROC curve in the present study, indicating better discriminatory ability than PCT and P-LCR. In contrast, PCT showed relatively high sensitivity but poor specificity, suggesting that it is more useful as an adjunctive marker than as an independent diagnostic test. Similarly, P-LCR demonstrated only modest diagnostic performance despite significantly higher mean values in the hyperdestructive group. These findings suggest that combining platelet indices with clinical assessment and routine laboratory investigations may improve diagnostic accuracy rather than relying on any single parameter alone [14].

An additional finding of the present study was the moderate positive correlation between platelet count and PCT, whereas MPV and PDW showed only weak correlations and P-LCR showed no significant correlation. Because PCT reflects total circulating platelet mass, its relationship with platelet count is expected. However, its relatively limited specificity observed in this study indicates that platelet mass alone cannot reliably distinguish the underlying mechanism of thrombocytopenia [15].

The clinical significance of these findings lies in the widespread availability of platelet indices as part of routine complete blood count analysis. Since these parameters require no additional blood sample, laboratory procedure, or cost, they can serve as useful initial screening tools, particularly in resource-limited settings where bone marrow examination may not be immediately feasible. Elevated MPV and PDW should increase suspicion for hyperdestructive thrombocytopenia, whereas relatively lower values may support further evaluation for hypoproductive disorders. However, platelet indices should be interpreted in conjunction with clinical findings, peripheral blood smear examination, and other laboratory investigations rather than being used as standalone diagnostic tests [16].

The present study has several strengths. It evaluated four routinely available platelet indices in a single cohort using standardized automated hematology analyzers and included patients with diverse etiologies encountered in routine clinical practice. Furthermore, diagnostic cut-off values were established using ROC curve analysis, providing clinically applicable information for differentiating hypoproductive and hyperdestructive thrombocytopenia.

Limitations

This study has several limitations. First, it was conducted at a single tertiary care center, which may limit the generalizability of the findings. Second, the sample size, particularly for the hypoproductive thrombocytopenia group, was relatively small. Third, platelet indices were measured using specific automated hematology analyzers, and the optimal cut-off values identified in this study may not be directly applicable to other analyzer platforms. Finally, because of the cross-sectional study design, causal relationships could not be established. Larger multicenter studies with standardized analyzer platforms are required to validate these findings and establish universally applicable diagnostic cut-off values.

## Conclusions

Platelet indices, particularly mean platelet volume (MPV) and platelet distribution width (PDW), demonstrated good diagnostic performance in differentiating hyperdestructive from hypoproductive thrombocytopenia. These routinely available parameters, obtained as part of the complete blood count, may serve as useful adjunctive tools in the initial evaluation of thrombocytopenic patients and help prioritize further diagnostic investigations, especially in resource-limited settings. However, platelet indices should be interpreted in conjunction with clinical findings and other laboratory investigations rather than as standalone diagnostic tests. Larger multicenter studies are warranted to validate these findings and establish standardized diagnostic cut-off values across different populations.
